# Diversity of Astroglial Effects on Aging- and Experience-Related Cortical Metaplasticity

**DOI:** 10.3389/fnmol.2018.00239

**Published:** 2018-07-13

**Authors:** Ulyana Lalo, Alexander Bogdanov, Yuriy Pankratov

**Affiliations:** ^1^School of Life Sciences, University of Warwick, Coventry, United Kingdom; ^2^Institute for Chemistry and Biology, Immanuel Kant Baltic Federal University, Kaliningrad, Russia

**Keywords:** ATP release, D-serine, caloric restriction, exocytosis, metaplasticy, CB1 receptors, BCM model

## Abstract

Activity-dependent regulation of synaptic plasticity, or metaplasticity, plays a key role in the adaptation of neuronal networks to physiological and biochemical changes in aging brain. There is a growing evidence that experience-related alterations in the mechanisms of synaptic plasticity can underlie beneficial effects of physical exercise and caloric restriction (CR) on brain health and cognition. Astrocytes, which form neuro-vascular interface and can modulate synaptic plasticity by release of gliotransmitters, attract an increasing attention as important element of brain metaplasticity. We investigated the age- and experience-related alterations in astroglial calcium signaling and stimulus-dependence of long-term synaptic plasticity in the neocortex of mice exposed to the mild CR and environmental enrichment (EE) which included ad libitum physical exercise. We found out that astrocytic Ca^2+^-signaling underwent considerable age-related decline but EE and CR enhanced astroglial signaling, in particular mediated by noradrenaline (NA) and endocannabinoid receptors. The release of ATP and D-Serine from astrocytes followed the same trends of age-related declined and EE-induced increase. Our data also showed that astrocyte-derived ATP and D-Serine can have diverse effects on the threshold and magnitude of long-term changes in the strength of neocortical synapses; these effects were age-dependent. The CR- and EE-induced enhancement of astroglial Ca^2+^-signaling had more stronger effect on synaptic plasticity in the old (14–18 months) than in the young (2–5 months) wild-type (WT) mice. The effects of CR and EE on synaptic plasticity were significantly altered in both young and aged dnSNARE mice. Combined, our data suggest astrocyte-neuron interactions are important for dynamic regulation of cortical synaptic plasticity. This interaction can significantly decline with aging and thus contributes to the age-related cognitive impairment. On another hand, experience-related increase in the astroglial Ca^2+^-signaling can ameliorate the age-related decline.

## Introduction

Although an age-related cognitive decline is widely recognized a major societal and scientific problem, fundamental mechanisms of brain longevity are not fully understood. Synaptic plasticity enables the mammalian brain to adapt to environmental challenges during development, adulthood and aging. Nowadays the age-related change in the cognitive functions is viewed not as complete loss of synaptic plasticity but as alteration of its mechanisms (Hillman et al., 2008; Nithianantharajah and Hannan, 2009; van Praag, 2009; Mercken et al., 2012; Merzenich et al., 2014).

Importantly, neural networks and synapses are remarkably responsive to environmental stimuli, physiological modifications, and experience (Hillman et al., 2008; Nithianantharajah and Hannan, 2009; van Praag, 2009; Mercken et al., 2012; Rodríguez et al., 2013; Merzenich et al., 2014). Physical exercise and environmental enrichment (EE) can have beneficial effects on aging brain, both in animal models and human patients (Hillman et al., 2008; Nithianantharajah and Hannan, 2009; van Praag, 2009; Mercken et al., 2012; Merzenich et al., 2014). Also, caloric restriction (CR), usually defined as a reduced intake of calories not causing malnutrition, can have life-extending effects, linked to improvement of brain health and plasticity (Mercken et al., 2012; Park et al., 2012; Madeo et al., 2014; López-Otín et al., 2016).

Aging- and experience-related alterations in synaptic plasticity are closely linked to brain metaplasticity, which is usually defined as “the plasticity of synaptic plasticity.” Metaplasticity can occur when priming synaptic or cellular activity or inactivity leads to persistent change in the direction or degree of synaptic plasticity (Abraham and Bear, 1996; Hulme et al., 2013a). Astrocytes are gaining an increasing attention as a very important element of brain cellular networks regulating metaplasticity (Hulme et al., 2013b; Monai et al., 2016; Boué-Grabot and Pankratov, 2017; Singh and Abraham, 2017). Astrocytes form interface between the synapses and brain vasculature (Gourine et al., 2010; Halassa and Haydon, 2010; Araque et al., 2014) and therefore are strategically positioned to couple the enriched mental and physical activity to the brain longevity. Astrocytes can also respond to both high-fat and calorie-restricted diet (Seidel et al., 2006; Lin et al., 2014; Metna-Laurent and Marsicano, 2015).

Importantly, astrocytes can exert bi-directional effects on synaptic plasticity by releasing different gliotransmitters (Pascual et al., 2005; Henneberger et al., 2010; Araque et al., 2014; Pougnet et al., 2014; Rasooli-Nejad et al., 2014; Pankratov and Lalo, 2015; Lalo et al., 2016; Boué-Grabot and Pankratov, 2017; Papouin et al., 2017a). It is conceivable that overall effect of astroglia on plasticity of particular type of synapses would depend on physiological (or pathological) context, i.e., pattern of local neural activity, repertoire of transmitters released from neurons and repertoire of post- and pre-synaptic receptors expressed in the synapse. Such dependence of astroglial modulation of synaptic plasticity on prior activity of network can render an important role for astrocytes in metaplasticity (Hulme et al., 2013b; Monai et al., 2016; Boué-Grabot and Pankratov, 2017; Singh and Abraham, 2017).

The responsiveness of synaptic plasticity to EE, physical activity and CR provides an opportunity to ameliorate the negative consequences of aging on cognitive function. Still, fundamental cellular and molecular mechanisms underlying the impact of CR and exercise on brain metaplasticity remain largely unexplored. There are also many uncertainties in the mechanisms of neuro-glial interaction (Bazargani and Attwell, 2016; Papouin et al., 2017b; Singh and Abraham, 2017; Fiacco and McCarthy, 2018; Savtchouk and Volterra, 2018), particularly in the aging brain (Rodríguez et al., 2014; Verkhratsky et al., 2014, 2017). Until recently, most studies of brain aging have been focused on functional changes in neural networks and alterations in neuronal morphology and gene expression whereas glia-neuron interactions remained largely overlooked. Recent reports of aging-related changes of Ca^2+^ -signaling, morphology and gene expression in astrocytes (Lalo et al., 2011, 2014b; Rodríguez et al., 2014; Verkhratsky et al., 2014, 2017; Soreq et al., 2017) highlighted a crucial importance of study of brain aging and neurodegeneration in the context of complex cellular interactions which maintain synaptic dynamics and homeostasis (De Strooper and Karran, 2016). Still, changes in the glia-driven modulation of synaptic metaplasticity in aging brain remain almost unexplored.

In the present article, we explored role for astroglial Ca^2+^-signaling and release of gliotransmitters in aging- and environment-related cortical metaplasticity. As a model of impaired astroglial exocytosis, we used dnSNARE mice whose validity, in particular the lack of neuronal expression of dnSNARE, has been recently verified (Pankratov and Lalo, 2015; Sultan et al., 2015; Lalo et al., 2016).

## Materials and Methods

All animal work has been carried out in accordance with UK legislation (ASPA) and “3R” strategy; all experimental protocols were approved by University of Warwick Ethical Review Committee and Animal Welfare Committee.

### Slice and Cell Preparation

Mice of two aged groups, 2–5 (average 3.3) months and 14–18 (average 16.8) months were anesthetized by halothane and then decapitated, in accordance with UK legislation. Brains were removed rapidly after decapitation and placed into ice-cold physiological saline containing (mM): NaCl 130, KCl 3, CaCl_2_ 0.5, MgCl_2_ 2.5, NaH_2_PO_4_ 1, NaHCO_3_ 25, glucose 15, pH of 7.4 gassed with 95% O_2_–5% CO_2_. Transverse slices (280 μm) were cut at 4^o^ C and then placed in physiological saline containing (mM): NaCl 130, KCl 3, CaCl_2_ 2.5, MgCl_2_ 1, NaH_2_PO_4_ 1, NaHCO_3_ 22, glucose 15, pH of 7.4 gassed with 95% O_2_–5% CO_2_ and kept for 1–4 h prior to cell isolation and recording.

Astrocytes were identified by their morphology under DIC observation, EGFP fluorescence (astrocytes from dn-SNARE and GFAP-EGFP mice) or staining with sulforhodamine 101 (astrocytes from WT mice). After recording, the identification of astrocyte was confirmed via functional properties (high potassium conductance, low input resistance, strong activity of glutamate transporters) as described previously (Lalo et al., 2011, 2014a; Rasooli-Nejad et al., 2014; Pankratov and Lalo, 2015).

### Electrophysiological Recordings

Whole-cell voltage clamp recordings from cortical neurones and astrocytes cells were made with patch pipettes (4–5 MΩ) filled with intracellular solution (in mM): 110 CsCl, 10 NaCl, 10 HEPES, 5 MgATP, 1 D-Serine, 0.1 EGTA, pH 7.35; Currents were monitored using an MultiClamp 700B patch-clamp amplifier (Axon Instruments, USA) filtered at 2 kHz and digitized at 4 kHz. Experiments were controlled by Digidata1440A data acquisition board (Axon Instruments, USA) and WinWCP software (Strathclyde University, UK); data were analyzed by self-designed software. Liquid junction potentials were compensated with the patch-clamp amplifier. The series and input resistances were respectively 5–7 MΩ and 600–1100 MΩ; both series and input resistance varied by less than 20% in the cells accepted for analysis.

Field excitatory postsynaptic potentials (fEPSPs) were measured via a glass micropipette filled with extracellular solution (0.5–1 MΩ resistance) placed in neocortical layer II/III. The fEPSPs were evoked by the stimulation of neuronal afferents descending from layers IV–V. For activation of synaptic inputs, axons originating from layer IV–VI neurons were stimulated with a bipolar coaxial electrode (WPI, USA) placed in layer V close to the layer IV border, approximately opposite the site of recording; stimulus duration was 300 μs. The stimulus magnitude was set 3–4 times higher than the minimal stimulus necessary to elicit a response in layer II pyramidal neurons (Rasooli-Nejad et al., 2014; Pankratov and Lalo, 2015; Lalo et al., 2016).

The long-term potentiation/depression (LTP/LTD) was induced by different number of trains of high-frequency theta-burst stimulation (HFS-trains); each train (100 ms-long) consisted of 10 pulses stimulated at 100 Hz, trains were delivered with 200 ms intervals, every 10 trains were separated by 10 s-long intervals.

### Multi-photon Fluorescent Ca^2+^-Imaging in Astrocytes

To monitor the cytoplasmic free Ca^2+^concentraton ([Ca^2+^]_i_) *in situ*, astrocytes of neocortical slices were loaded via 30 min incubation with 1 μM of Rhod-2AM or Oregon Green Bapta-2AM and sulphorhodamine 101 (wild-type (WT) mice) at 33°C. Two-photon images of neurons and astrocytes were acquired at 5 Hz frame-rate using a Zeiss LSM-7MP multi-photon microscope coupled to a SpectraPhysics MaiTai pulsing laser; experiments were controlled by ZEN LSM software (Carl Zeiss, Germany). Images were further analyzed offline using ZEN LSM (Carl Zeiss) and ImageJ (NIH) software. The [Ca^2+^]_i_ levels were expressed as ΔF/F ratio averaged over a region of interest (ROI). For analysis of spontaneous Ca^2+^–transients in astrocytes, three ROIs located over dendrites and one ROI located over the soma were chosen. Overall Ca^2+^-response to receptors agonists or synaptic stimulation was quantified using an ROI covering the whole cell image.

### Measurement of Extracellular Concentration of ATP and D-Serine in the Brain Tissue

The concentration of ATP within cortical slices was measured using microelectrode biosensors obtained from Sarissa Biomedical Ltd (Coventry, UK). A detailed description of the properties of biosensors and recording procedure has been published previously (Frenguelli et al., 2007). Briefly, biosensors consisted of ATP or D-Serine metabolizing enzymes immobilized within a matrix on thin (25 μM) Pt/Ir wire. This allowed insertion of the sensors into the cortical slice and minimized the influence of a layer of dead surface tissue. ATP and D-serine biosensors were used simultaneously. A third, null, biosensor was also used. This sensor is identical to the ATP and D-serine sensors and has a matrix, but lacks enzymes. The signal from the null sensor was subtracted from the signal obtained on the ATP and D-serine sensor. This allows the contribution of any non-specific electroactive substances that bypass the sensor screening layer to be eliminated. Biosensors show a linear response to increasing concentration of ATP and D-Serine and have a rise time less than 10 s (Frenguelli et al., 2007). Biosensors were calibrated with known concentrations (10 μM) of ATP and D-Serine before the slice was present in the perfusion chamber and after the slice had been removed. This allowed compensation of any reduction in sensitivity during the experiment. The integrity of the screening layer was assessed with 10 μM 5-HT. Biosensor signals were acquired at 1 kHz with a 1400 CED interface and analyzed using Spike 6.1 software (Cambridge Electronics Design, Cambridge, UK).

### Data Analysis

All data are presented as mean ± SD and the statistical significance of differences between data groups was tested by two-tailed unpaired *t*-test, unless indicated otherwise. For all cases of statistical significance reported, the statistical power of the test was 0.8–0.9. Each neocortical slice was used only for one experiment (e.g., fluorescent recordings in single astrocyte or single LTP induction experiment). The number of experiments/cells reported is therefore equal to the number of slices used. The experimental protocols were allocated randomly so the data in any group were drawn from at least from four animals, typically from 5 to 12 mice. The average ratio of experimental unit per animal was 1.3 for the LTP experiments and 1.5 for biosensor and fluorescent Ca^2+^-measurements.

The spontaneous transmembrane currents recorded in neurons were analyzed offline using methods described previously (Lalo et al., 2011, 2016). The amplitude distributions of spontaneous and evoked currents were analyzed with the aid of probability density functions and likelihood maximization techniques; all histograms shown were calculated as probability density functions. The amplitude distributions were fitted with either multi-quantal binomial model or bi-modal function consisting of two Gaussians with variable peak location, width and amplitude. Parameters of models were fit using likelihood maximization routine.

## Results

### Age- and Environment-Related Alterations in Astroglial Ca^2+^-Signaling

Previously, we have reported the aging-related decline in the density of purinergic and glutamatergic receptors and their contribution to Ca^2+^-signaling in neocortical astrocytes (Lalo et al., 2011, 2014b). In accordance with decrease in the Ca^2+^-signaling, the exocytosis of gliotransmitters, such ATP, D-Serine and Glutamate, from neocortical astrocytes also declined with aging (Lalo et al., 2011, 2014b; Lalo and Pankratov, 2017). In the present work, we tried to elucidate whether EE or CR can mitigate the negative effects of aging. We have focused primarily on receptors for NA and endocannabinoids since recent results highlighted the importance of these receptors for glia-neuron communications (Min and Nevian, 2012; Ding et al., 2013; Paukert et al., 2014; Metna-Laurent and Marsicano, 2015; Pankratov and Lalo, 2015; Oliveira da Cruz et al., 2016). In particular, our data (Rasooli-Nejad et al., 2014; Pankratov and Lalo, 2015) have shown that both α1-adrenoreceptors and CB1 receptors can trigger release of gliotransmitters from neocortical astrocytes.

**Figure 1:**
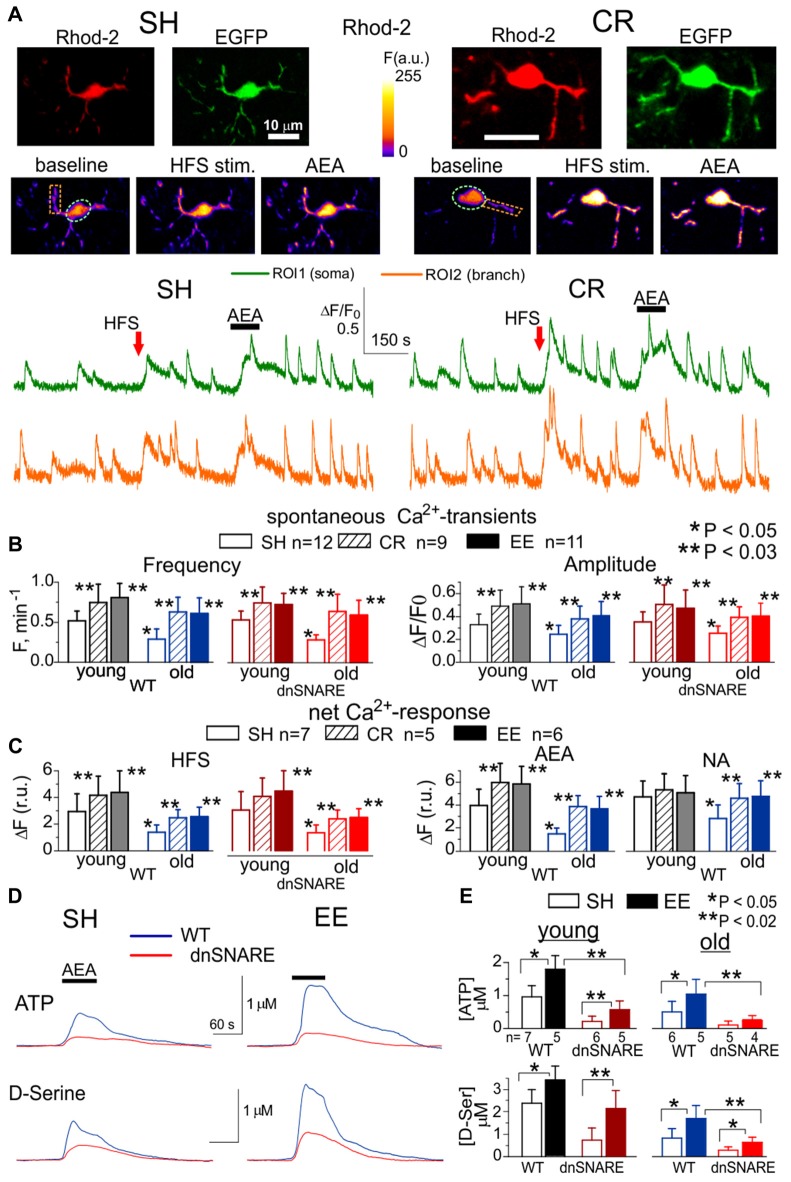
Age- and experience-related changes in the adrenergic Ca^2+^ signaling and release of gliotransmitters in the neocortex. Astroglial Ca^2+^ -signaling **(A–C)** and release of ATP and D-Serine were evaluated in the neocortex of 2–5 month-old (young) and 14–18 month-old mice (old) as described previously in Lalo et al. (2014a), Rasooli-Nejad et al. (2014) and Pankratov and Lalo (2015). The dnSNARE mice and their wild-type (WT) littermates were kept either in standard housing (SH) or exposed to environmental enrichment (EE) or caloric restriction (CR) as described in the text. **(A)** Representative multi-photon images of EGFP fluorescence and presudo-color images of Rhod-2 fluorescence recorded in the astrocytes of old dn-SNARE mouse before and after the 100 ms-long episode of high-frequency stimulation (HFS) of cortical afferents and application of CB1 receptor agonist anandamide (AEA). Graphs below show the time course of Rhod-2 fluorescence averaged over regions indicated in the fluorescent images. Note the increase in the spontaneous in the Ca^2+^ -elevations and responses to HFS and application of AEA. **(B)** The pooled data on peak amplitude and frequency of the baseline spontaneous Ca^2+^- transients recorded in astrocytes of WT and dn-SNARE mice of different age and treatment groups. Number and size of spontaneous events were pooled for the whole cell image. **(C)** The pooled data on the net responses to the HFS and application of AEA (500 nM) and noradrenaline (NA, 1 μM). Net response was evaluated as an integral Ca^2+^-signal measured within 3 min after stimulation, averaged over the whole cell image and normalized to the baseline integral Ca^2+^ signal. Data in the panels **(B,C)** shown as mean ± SD for the number of cells indicated. Note the lack of the difference in the Ca^2+^-signaling in the WT and dnSNARE mice. Asterisks (*,**) indicate statistical significance of the effect of EE- or CR-treatment (as compared to SH) and difference between the old and young mice of the same treatment group. **(D,E)** AEA-activated release of ATP and D-Serine in the neocortical slices of SH and EE mice was detected using microelectrode sensors as described previously (Lalo et al., 2014a,b; Rasooli-Nejad et al., 2014). **(E)** The representative responses of cortical slices of the old WT and dn-SNARE mice to the application of 500 nM AEA were recorded using microelectrode sensors to ATP and D-Serine placed in the layer II/III. The data are shown as an elevation relative to the resting concentration. **(E)** The pooled data on the peak magnitude of ATP- and D-Serine transients evoked by application of AEA; data shown as mean ± SD for number of experiments indicated. Asterisks (*,**) indicate statistical significance of difference in the magnitude of ATP- and D-serine responses between WT and dn-SNARE mice (unpaired *t*-test) and SH- and EE-mice of similar genotype. The significant reduction in the AEA-evoked responses in the cortical slices from dn-SNARE mice strongly supports the vesicular mechanism of ATP and D-Serine release from astrocytes. Note the decrease in the ATP- and D-Serine transients in the old mice and EE-induced increase.

We explored the difference in the spontaneous and synaptically-evoked cytosolic Ca^2+^-transients in the neocortical layer 2/3 astrocytes of 2–4 months old (young adults) and 14–18 months old (old) WT and dnSNAREs mice. Astroglial Ca^2+^ signaling was monitored using multi-photon fluorescent microscopy as described previously (Lalo et al., 2014a; Rasooli-Nejad et al., 2014; Pankratov and Lalo, 2015). We compared animals kept under standard housing conditions (SH) vs. animals exposed to the EE from birth (Correa et al., 2012), including *ad libitum* access to running wheel, or kept on mild CR(CR) diet (food intake individually regulated to maintain the body weight loss of 10%–15%) for 4–6 weeks. We also assessed the impact of exogenous activation of adrenergic and eCB receptors on astroglial Ca^2+^ signaling under these conditions (Figures 1A,B).

There was no significant difference in the astroglial Ca^2+^-signaling between dnSNARE mice and their WT littermates. In both WT and dnSNARE mice of SH, the amplitude and frequency of spontaneous Ca^2+^-transients in the neocortical astrocytes underwent significant decrease with aging. The EE and CR had significant positive effect on the astroglial Ca^2+^-signaling both in the young and old mice (Figures 1B,C). Interestingly, effects of EE and CR on the amplitude and frequency of Ca^2+^-transients in the old age (60%–95% and 70%–110% correspondingly) were more profound than in the young mice (35%–50% and 30%–45%).

To probe the responses of astrocyte to the stimulation of neighboring synapses, we evaluated astrocytic Ca^2+^-transients evoked by the short episode of high-frequency stimulation (HFS) of thalamo-cortical afferents, as described previously (Lalo et al., 2011, 2016; Rasooli-Nejad et al., 2014). The HFS-evoked Ca^2+^-responses followed the similar trends as spontaneous astroglial activity: significant reduction in the old age was opposed by EE- and CR-induced increase (Figure 1C).

Similarly to our previous reports (Rasooli-Nejad et al., 2014; Pankratov and Lalo, 2015), activation of CB1 receptors by 500 nM anandamide (AEA) or α1-adrenoreceptors by 1 μM NA evoked profound elevation in the cytosolic Ca^2+^ concentration in the astrocytes of young but not old mice (Figures 1A,D). The EE and CR had a moderate effect on the amplitudes of NA- and AEA-evoked responses in the young mice but caused the significant enhancement of astroglial responses in the old mice (Figure 1D).

Apart from direct activation of Ca^2+^-responses, astroglial CB1 receptors and α1-adrenoreceptors are capable to enhance the spontaneous signaling, as reported previously (Min and Nevian, 2012; Rasooli-Nejad et al., 2014; Pankratov and Lalo, 2015). Indeed, application of NA or AEA caused significant increase in the amplitude and frequency of spontaneous Ca^2+^-transients in the astrocytes of mice of SH (Figure 1C). Rather surprisingly, the relative effects of both NA and AEA were much smaller in the EE-mice or CR-mice. This was most likely related to the higher baseline spontaneous activity in astrocytes of EE- and CR-mice as compared to SH.

To evaluate the age-related alterations in gliotransmission, we measured extracellular concentrations of ATP and D-Serine in the neocortical tissue of WT and dnSNARE mice with microelectrode biosensors and activated astrocytes with AEA. In the old age and environment groups, the CB1 receptor-activated elevations in the ATP and D-Serine concentrations were significantly reduced in the dnSNARE mice, supporting the previous data (Lalo et al., 2014a,b; Rasooli-Nejad et al., 2014) on important contribution of astroglial exocytosis in the release of these transmitters. Consistent with changes in the Ca^2+^-signaling, release of ATP and D-Serine significantly decreased in the old age but was strongly up-regulated by the EE (Figures 1D,E). These results closely agree with our previous data on adrenoceptor-activated release of gliotransmitters (Lalo and Pankratov, 2017).

**Figure 2:**
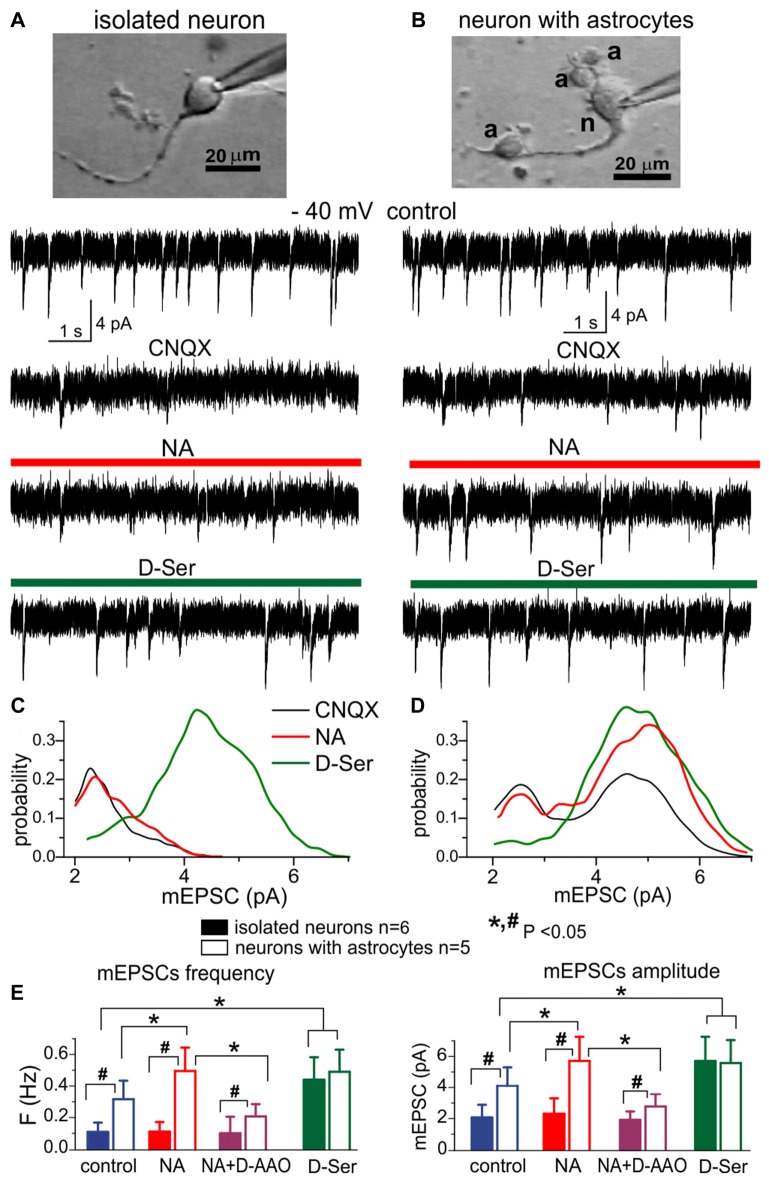
The lack of efficient release of D-Serine from neurons.** (A,C)** Modulation of NMDA receptors by the D-Serine was evaluated in acutely isolated neocortical neurons retaining the functional glutamatergic synapses (Lalo et al., 2006; Lalo and Pankratov, 2017). **(B,D)** Similar recordings were made in the neuron attached to few astrocytes (marked as **“a”**) after dissociation of this neuron-astrocyte “bundle” from neocortex of WT mice; identification of astrocytes was confirmed by their electrophysiological properties after recordings (Lalo et al., 2014a; Rasooli-Nejad et al., 2014; Lalo and Pankratov, 2017). **(A,B)** Representative whole-cell currents recorded at −40 mV in the presence of picrotoxin, TTX, PPADS and 5-BDBD (control) before and after consecutive application of CNQX, (NA, 1 μM) and exogenous D-Serine (10 μM) after washout of NA. The transient events which can be seen in the neurons **(A,B)** in the control are miniature excitatory synaptic currents, as demonstrated in Lalo et al. (2006) and Lalo and Pankratov (2017). Note that amplitude and frequency of the events recorded in the control in fully isolated neurone are similar to the dissociated neurone with attached astrocytes **(B)**. Inhibition of AMPA receptors with CNQX markedly suppressed the spontaneous activity in the isolated neuron as compared to its counterpart with attached astrocytes **(B)**. The CNQX–insensitive currents were completely eliminated by application D-APV in both cases confirming they were mediated by NMDARs (data are not shown). **(C,D)** The corresponding amplitude distributions (probability density functions) for the spontaneous NMDAR currents recorded in neurons shown in **(A,B)**. **(E)** Pooled data (mean ± SD for number of cells indicated) on the amplitude and frequency of NMDAR-mediated mEPSCs recorded in fully isolated neurons (open bars) and dissociated neurons with attached astrocytes (closed bars) at −40 mV in presence of CNQX (control) and after application of NA alone, NA in presence of D-amino acid oxidase (DAAO, 0.15 U/ml) or exogenous D-Serine. The statistical significance (un-paired *t*-test) of the difference between fully isolated neurons and neurons with astrocytes is indicated by the hash symbol (#); asterisks (*) indicate statistically significance of the effect of NA and D-Serine in comparison to control conditions (−40 mV, CNQX). Note that the amplitude and frequency of NMDAR-mediated mEPSCs in fully isolated neurons were much lower in comparison to the dissociated neuron with astrocytes **(A,E)**. However, mEPSCs in the isolated neurons were restored in the presence of exogenous D-Serine, as evidenced by appearance of events with larger amplitude **(C)**. The most straightforward explanation is that most NMDA receptors on the membrane of fully isolated neurons were not exposed to sufficient concentrations of co-agonist until exogenous D-Serine was applied. Application of NA led to increase in the number of mEPSCs with larger amplitudes **(D)** in the neurons with attached astrocytes but not in fully isolated neurons, suggesting that effect of NA on synaptic transmission is mediated by release of gliotransmitters. The effect of NA on the amplitude and frequency of mEPSCs was strongly attenuated by D-AAO, suggesting the involvement of D-Serine.

Our results suggest a major role of astrocytes in the release of D-Serine, which is in line with numerous previous reports (Panatier et al., 2006; Henneberger et al., 2010; Sultan et al., 2015; Papouin et al., 2017b). Since the importance and specificity of glial release of D-Serine is hotly debated currently (Wolosker et al., 2016; Papouin et al., 2017b; Savtchouk and Volterra, 2018), we tried to directly assess contribution of neurons and astrocytes in the release of D-serine. For this purpose, we used acutely-isolated neocortical neurons which were devoid of the influence of glial cells (Figure 2). We used non-enzymatic vibro-dissociation which retains functional synapses on the dendrites of isolated neurons, which can be verified by the presence of miniature spontaneous synaptic currents (Duguid et al., 2007; Rasooli-Nejad et al., 2014; Lalo et al., 2016; Lalo and Pankratov, 2017). Apart from the pure isolated neurons, the vibro-dissociation technique allows, upon some adjustment, to dissociate neurons with few astrocytes attached. Such neuron-astrocyte “bundle,” retaining a certain proportion of intimate contacts between astrocytic and neuronal membranes, could serve as a “minimalistic” model of glia-neuron interaction unit (Rasooli-Nejad et al., 2014; Lalo and Pankratov, 2017).

We recorded the NMDA receptor-mediated spontaneous currents (NMDAR mEPSCs) in the acutely-dissociated neocortical pyramidal neurons at membrane potential of −40 mV in the presence of 100 μM picrotoxin, 30 μM CNQX and 20 μM PPADS (Figures 2A,B). To compensate for the putative depletion of neuronal D-Serine content due to intracellular perfusion, intracellular solution was supplemented with 1 mM D-Serine; intracellular concentration of EGTA was set to 0.1 mM. In the absence of external D-Serine or glycine, fully isolated neurons exhibited a low baseline frequency (0.11 ± 0.04 Hz, *n* = 6) of NMDAR-mediated mEPSCs and the application of NA did not cause notable changes in their amplitude or frequency (Figures 2A,E). Application of exogenous D-Serine dramatically increased the average amplitude and notably increased the frequency of mEPSCs. This was accompanied by the shift of mEPSCs amplitudes (Figure 2C) towards higher quantal size (from 2.3 ± 0.7 pA to 4.4 ± 1.2 pA, *n* = 6). In contrast to the fully isolated neurons, the NMDAR mEPSCs recorded in the “neuron-astrocyte bundles,” could be observed at relative high frequency (0.32 ± 0.11 Hz) even in the absence of exogenous D-Serine (Figure 2B). They had bimodal amplitude distributions with peaks at 2.4 ± 0.8 pA and 4.7 ± 1.4 pA (*n* = 5). Activation of astrocytes by NA cause a significant increase both in the mean amplitude and frequency of mEPSCs in “neuron-astrocyte bundles” (correspondingly 49 ± 24% and 77 ± 38%, *n* = 5); which was accompanied by increase in the number of mEPSCs of larger quantal size (Figure 2D). Importantly, the NA-induced facilitation of mEPSCs was efficiently blocked by the application of exogenous D-amino acid oxidase (Figure 2E), suggesting an involvement of D-Serine in the action of NA. The effect of exogenous D-Serine on NMDAR mEPSCs in neurons with attached astrocytes did not differ considerably from the fully isolated neurons (Figures 2D,E).

The most parsimonious explanation of the above results would be a lack of D-Serine (or other co-agonists) in the vicinity of glutamatergic synapses devoid astroglial influence, which drives the amplitude of NMDAR mEPSCs below the threshold of detection. Activation of astrocytes by NA triggers release of D-serine, which diffuses to nearby synapses where it enhances the NMDAR mEPSCs. Application of exogenous D-Serine restores the amplitudes of NMDAR-mediated currents in majority of synapses. Our results argue against the predominant role of neurons in release of D-Serine (Wolosker et al., 2016) and agree with recent transcriptomic data on the expression of serine racemase (*SRR*) gene in cortical astrocytes (Chai et al., 2017) and *in vivo* data on the physiological role for glial-derived D-Serine (Papouin et al., 2017a,b).

Combined, the above results demonstrate that spontaneous and evoked Ca^2+^-signaling in astrocytes and release of gliotransmitters can undergo significant decrease with aging but can be enhanced by EE or CR. It is also worth to note that astroglial CB1 and adrenergic receptors were capable to activate Ca^2+^-elevation and release of gliotransmitters even in old mice of SH supporting the notion that glia-neuron communications do not stop with aging. Our data also show a common trend of EE and CR to enhance astroglial signaling mainly in case when it is “weakened,” e.g., in the old mice and suggest an existence of “optimal” level of astroglial Ca^2+^-signaling which may be reached in the younger age.

### Impact of Astrocytes on Synaptic Plasticity in the Neocortex

Role for heterosynaptic cortical metaplasticity in brain computation, learning and memory is often studied in the framework of BCM-model (Bienenstock et al., 1982) whose two essential prerequisites are: bidirectional synaptic modification and sliding modification threshold. Accordingly, the general paradigm of BCM model, sub-threshold stimulation should induce long-term depression (LTD) whereas more stronger stimuli should induce potentiation (LTP) and prior stimulation (experience) can shift the LTD/LTP threshold one way or another (Bienenstock et al., 1982; Hulme et al., 2013b).

**Figure 3:**
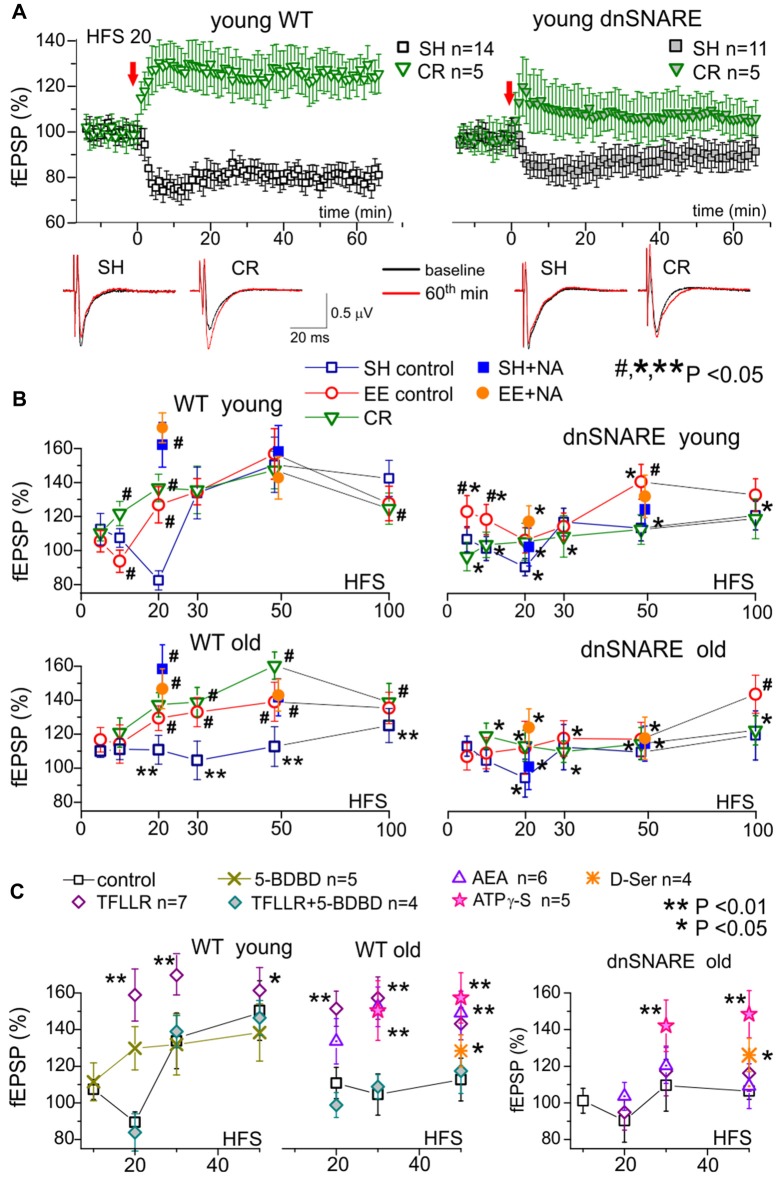
Impact of gliotransmission on synaptic plasticity in the neocortex. Long-term potentiation (LTP) of field excitatory postsynaptic potentials (fEPSPs) was induced in the layer 2/3 of somatosensory cortex of the WT and dnSNARE mice by different number of pulses of high-frequency theta-burst stimulation (100 ms-long trains of 100 Hz pulses delivered with 200 ms intervals, every 10 trains were separated by 10 s-long intervals), as previously described (Lalo et al., 2014a, 2016; Rasooli-Nejad et al., 2014; Pankratov and Lalo, 2015). **(A)** The representative time course of changes in the fEPSP induced by 20 HFS trains delivered at zero time, measured in the young mice of SH and mice exposed to CR. Dots in the graphs represent the average of six consecutive fEPSPs; data are shown as mean ± SD for number of experiments indicated. Data were normalized to the fEPSP slope averaged over 10 min period prior to the HFS. The insets show the average fEPSP waveforms recorded before and 60 min after the HFS. Note the difference in the effect of CR on long-term depression (LTD)/LTP switch in the WT and dnSNARE mice. **(B)** The stimulus-dependence of the long-term changes in the neocortical fEPSP in the old mice of different genotypes at different conditions. Graphs show the magnitude of LTP/LTD evaluated as relative increase in the fEPSP slope at 60th min, averaged across 10 min time window and plotted against the number of HFS trains delivered. Each data point shows mean ± SD for the following number of experiments/mice used): 10–22 (8–15) for SH, 6–9 (5–6) for EE and 6–7 (4–5) for CR mice. The graphs also show the effect of activation of astrocytes with 1 μM NA [REF] on the LTP induced by 20 and 50 HFS trains; NA was applied for 5 min 2 min prior to HFS. The statistical significance (2-population unpaired *t*-test) for the difference between genotypes, age- and treatment groups was indicated by different symbols as follows: (#)–effect of EE or CR as compared to SH mice of the same genotype and age and the effect of NA as compared to mice of same age, genotype and housing in the control; (*) dnSNARE mice vs. their WT littermates of the same age and housing; (**) old vs. young mice of the same genotype and housing. Note the different pattern of changes in the LTP/LTD between dnSNARE and WT mice and significant effects of EE and CR in the old WT mice. **(C)** The effects of astrocyte-derived ATP on neocortical synaptic plasticity in the young and old mice of SH was evaluated using the same experimental paradigm as in **(A,B)**. The release of gliotransmitters was activated by the agonists of astrocyte-specific PAR-1 receptors (Lalo et al., 2016) TFLLR (10 μM) in the control or after inhibition of neuronal P2 × 4 receptors to ATP with selective antagonist 5-BDBD (5 μM). Alternatively, enhancement of gliotransmitter release was activated via CB1 receptors (Rasooli-Nejad et al., 2014) or mimicked by exogenous application of ATP analog ATPγS (10 μM) or D-Serine (10 μM). All drugs were applied for 5 min 2 min before the LTP induction. Asterisks (* and **) indicate statistical significance of difference in the LTP magnitude as compared to the mice of same age and genotype under control conditions (unpaired *t*-test). Under control conditions, activation of astrocytes with TFLLR significantly increased the LTP in young and old WT but not the dnSNARE mice. Inhibition of P2×4 receptors prevented the TFLLR-induced enhancement of LTP. Contrary to TFLLR-activated release, application of exogenous gliotransmitters had significant effects both in the WT and dnSNARE mice. Combined, these results suggest the importance of astrocyte-derived ATP for modulation of synaptic plasticity in the neocortex.

There is accumulating evidence of both positive and negative effects of astrocytes on the strength of excitatory synapses mediated by glia-derived ATP and adenosine (Hulme et al., 2013b; Boué-Grabot and Pankratov, 2017). Furthermore, our recent data demonstrated that glia-derived ATP can cause down-regulation of NMDA receptors and affect the threshold of LTP induction (Lalo et al., 2016). This effect contrasts with widely accepted positive effect of glia-derived NMDA receptor co-agonist D-Serine on LTP (Henneberger et al., 2010; Papouin et al., 2017a). Hence, one could expect the gliotransmission to be important, if not quintessential, element of cortical metaplasticity. To test this hypothesis, we explored the stimulus-dependence of the LTP of fEPSPs in the neocortical layer 2/3 of WT and dnSNARE mice. The fEPSPs were evoked by the stimulation of neuronal afferents descending from layers IV-V (Rasooli-Nejad et al., 2014; Pankratov and Lalo, 2015; Lalo et al., 2016); LTP was induced by different number of pulses of high-frequency theta-burst stimulation (100 ms-long trains of 100 Hz pulses delivered with 200 ms intervals, every 10 trains were separated by 10 s-long intervals). Similarly to Ca^2+^-signaling experiments, we compared effects of EE and CR on LTP in the WT and dnSNARE mice (Figure 3).

Under control conditions, the long-term plasticity of fEPSPs in the young WT mice exhibited a characteristic stimulus-strength dependence: no potentiation at very weak stimulus, depression at moderate strengths and then potentiation which magnitude grew sharply upon reaching certain threshold but decline slightly in a bell-shaped manner at very strong stimuli. Such dependence goes very well in line with predictions of the BCM theory.

Importantly, impairment of glial exocytosis in the dnSNARE mice dramatically “flattened” the stimulus-dependence inhibiting both the depression at moderate stimuli and potentiation at strong stimuli (Figure 3B). The similar “flattened” pattern of LTP/LTD dependence on stimulus strength was observed in the old mice (Figure 3B). Taking into account the significant age-related decrease in astroglial Ca^2+^-signaling, one can suggest that decrease in the Ca^2+^-dependent release of gliotransmitters (either aging-related or due to dnSNARE expression) strongly attenuates the extent of bi-directional modulation of plasticity of neocortical synapses.

This hypothesis was corroborated by the effects of activation of astrocytes by exogenously applied NA (1 μM) and AEA (200 nM). Previously, we have verified that effects of these agonists on synaptic transmission and plasticity are predominantly related to their ability to activate the astroglial Ca^2+^-signaling and release of gliotransmitters (Lalo et al., 2014a, 2016; Rasooli-Nejad et al., 2014; Pankratov and Lalo, 2015). The 5 min-long application of NA (started 2 min prior to the induction protocol) significantly increased the magnitude of LTP induced in the neurons of younger WT mice by weak stimulation (20 HFS pulses) and the magnitude of LTP in older mice both at weak and strong (50 HFS pulses) stimuli. The NA-induced LTP enhancement was strongly attenuated in the dnSNARE mice supporting the importance of astroglial exocytosis for this effect. The similar action was shown by application of AEA or agonist of glia-specific PAR-1 receptors (TFLLR, 10 μM; Figure 3C).

These data closely agree with our previous results that by astroglial α1-ARs and CB1 receptors can facilitate LTP induction by triggering the release of ATP and D-Serine (Lalo et al., 2014a, 2016; Rasooli-Nejad et al., 2014; Pankratov and Lalo, 2015).

To verify that same molecular mechanisms can rescue the LTP in old mice, we investigated the effect of inhibition of ATP receptors and exogenous application of gliotransmitters (Figure 3C). First, selective inhibition of neuronal P2×4 receptors, which are not strongly expressed by neocortical astrocytes (Lalo et al., 2008), by 5-BDBD (5 μM) decreased both the magnitude and threshold of LTP; similar effects were recently observed in the P2×4 knock-out mice (Lalo et al., 2016). Second, inhibition of P2×4 receptors occluded the positive effects of AEA and TFLLR both in the young and old mice (Figure 3C). Finally, application of non-hydrolyzable ATP analog ATP-γS (10 μM) or D-Serine (10 μM) enhanced the LTP in the neocortex of both WT and dnSNARE mice.

Based on these results one could suggest that Ca^2+^ dependent release of ATP and D-Serine from astrocytes may be implicated in cortical metaplasticity by modifying both threshold and magnitude of long-term alterations of synaptic strength. Hence, aging-related decline in gliotransmission can lead to deficit in the LTP which could, in turn, be rescued by enhancement of astroglial signaling, for example by EE or CR (Figure 1). This notion was strongly supported by our observations of LTP modifications in the EE- and CR-exposed WT and dnSNARE mice (Figure 3B).

In the young WT mice, the main effect of EE and CR was a marked leftward shift in the threshold of LTP induction without increase in the maximal LTP amplitude (Figure 3B). So, both EE and CR caused statistically significant alterations in the LTP only at weaker stimuli (10 and 20 HSF). However, in the old WT mice, the EE- and CR-induced enhancement in astroglial signaling was accompanied by the leftward shift in the LTP induction threshold and significant increase in the magnitude of LTP at moderate and strong stimuli. Generally, the exposure of old mice to the EE and CR lead to a “younger” LTP phenotype. In the dnSNARE mice, both young and old, the positive action of CR on the LTP magnitude was effectively occluded strongly suggesting the crucial importance of release of gliotransmitters for CR-related metaplasticity. At the same time, dnSNARE expression had a less straightforward effect on the EE-induced modifications of LTP.

Although there was statistically significant difference in the LTP magnitude between WT and dnSNARE EE-exposed young mice, there also was significant EE-induce increase in the LTP in dnSNARE mice (as compared to SH mice) at most induction protocols. This rather surprising observation of “rescue” of LTP in the young dnSNARE mice by EE have two important implications. First, lack of LTP deficit in the EE dn-SNARE mice strongly argues against the non-selective “leaky” expression of dn-SNARE transgene in neurons. Second, the LTP deficit in the dn-SNARE mice kept in SH clearly demonstrated the physiological relevance of exocytotic release of gliotransmitters from astrocytes. The marked responsiveness of dn-SNARE mice to EE suggested the existence of some compensatory mechanisms which could overcome the deficit in vesicular gliotransmission. The most straightforward explanation might be a mosaic expression of dnSNARE transgene across the astrocyte population (Pascual et al., 2005; Sultan et al., 2015) and EE-induced enhancement of Ca^2+^-signaling and the release of gliotransmitters from non-dnSNARE astrocytes. In the neocortex and hippocampus, just 50%–60% of astrocytes express dnSNARE (Halassa et al., 2009). Due to the presence of significant proportion of un-affected astrocytes in the dnSNARE mice, the EE-induced enhancement of Ca^2+^-signaling can, very likely, lead to overall increase of gliotransmitter release even in dnSNARE animals (albeit in a lesser extent than in their WT littermates). Combined with threshold-like behavior of LTP, EE-induced increase in gliotransmitter release can be capable to rescue the LTP in dnSNARE mice. Also, impact of EE on synaptic transmission may involve other mechanisms of neuronal origin, like BDNF/MSK1-dependent homeostatic synaptic scaling (Correa et al., 2012).

We would like to emphasize that the pattern of EE-induced alterations in stimulus-dependence of LTP was different in the young dnSNARE and WT mice. The increase in the LTP both at weak and strong stimuli in the dnSNARE mice contrasted to the alterations of the threshold, but not in the maximal magnitude, in their WT littermates. Furthermore, the EE was not very efficient in the old dnSNARE mice (Figure 3B), except at very strong stimulus (100 HFS) suggesting that role of glial exocytosis can increase with age. Hence, one way or another, our data strongly suggest the involvement of glial exocytosis in the EE-induced metaplasticity.

Interestingly, the EE modified the effect of additional action of astrocytes on the LTP. Application of NA caused significant enhancement of LTP in the neocortex of WT mice only at weaker stimulus (20 HFS pulses) but did not have notable effect on the LTP induced by stronger stimulus (50 HFS) in both age groups. One could suggest that exposure to EE, causing increase in astrocytic Ca^2+^ -signaling and gliotransmission, “pre-emptied” the effect of activation of adrenoceptors.

Taken together, the above results strongly support the importance of astroglial Ca^2+^-signaling and gliotransmission for the bi-directional modulation of synaptic plasticity in the neocortex. Our data also suggest an importance of astroglial eCB and NA receptors for metaplasticity-related effects of aging, EE and CR.

## Discussion

During last two decades a plethora of experimental results has been obtained showing that various gliotransmitters can exert different, and sometimes opposing, effects on neuronal signaling (Gordon et al., 2009; Araque et al., 2014; Pougnet et al., 2014; Bazargani and Attwell, 2016; Boué-Grabot and Pankratov, 2017). In particular, release of glutamate and D-serine from astrocytes can enhance the activity of NMDA component of excitatory synapses and thereby the long-term synaptic plasticity (Panatier et al., 2006; Henneberger et al., 2010; Rasooli-Nejad et al., 2014). There is also an evidence of the positive impact of glia-derived ATP on activity and trafficking of AMPA receptors (Boué-Grabot and Pankratov, 2017). Our previous data showed that glia-derived ATP can down-regulate the GABAergic tonic and phasic inhibitory transmission (Lalo et al., 2014a). Hence, one might expect the glia-derived ATP to shift the balance between excitatory and inhibitory synaptic inputs towards excitation and thereby facilitate the induction of LTP. At the same time, our recent data have shown that ATP, acting via postsynaptic P2X receptors, can down-regulate NMDA receptors and decrease the magnitude of LTP (Lalo et al., 2016). Thus, release of ATP and D-Serine from astrocytes can lead to bi-directional modifications of synaptic strength and thereby play an important role in the mechanisms of brain metaplasticity. Our results present several lines of evidence to support this notion.

We observed that positive effects of EE and CR can counterbalance the aging-related decrease in the Ca^2+^-signaling and release of gliotransmitters from neocortical astrocytes. The EE- and CR-induced enhancement in astroglial function was accompanied by marked alterations in the stimulus-dependence of the neocortical LTP. These alterations different considerably in the WT and dnSNARE mice supporting the importance of release of gliotransmitters for the beneficial effects of EE and CR on synaptic plasticity in the old age. The EE- and CR-induced enhancement of the LTP in old mice could also be mimicked, irrespective of dnSNARE expression, by short-term application of ATP analog (ATPγS) and D-Serine during LTP induction period, indicating the importance of these gliotransmitters.

Our results also show that additional activation of astrocytes via eCB, adrenergic or PAR-1 receptors during LTP induction protocol can increase the magnitude of the resulting LTP. Importantly, such activation of astrocytes caused significant effect only in cases when LTP magnitude was small, i.e., at weaker stimuli in the younger mice of SH or at stronger stimuli in the old mice. The α1-AR mediated facilitation of LTP was also occluded by the treatments (EE and CR) which already increased astrocytic Ca^2+^-signaling and LTP.

Combined, our results suggest that activation of astrocytes performs a “balancing act,” facilitating the LTP in case of deficit in signaling and gliotransmission but attenuating the LTP magnitude in case of stronger stimuli and higher levels of astroglial activity. A strong dependence of glia-driven modulation, of both the threshold and magnitude of LTP, on the activity of neuronal P2×4 receptors (Figure 3, see also Lalo et al., 2016) may imply an important role for ATP receptor-mediated down-regulation of GABA and NMDA receptors in the mechanisms of metaplasticity.

Overall, our observations of LTP modifications in the EE- and CR-exposed WT and dnSNARE mice go in line with our hypothesis that Ca^2+^ dependent release of ATP and D-Serine from astrocytes can affect both threshold and magnitude of long-term alterations of synaptic strength and thereby can be very important for cortical metaplasticity. Our data also indicate that modulation of neocortical plasticity, in particular via glia-neuron communications, can be analyzed in the framework of BCM theory.

It becomes increasingly evident that net effect of activation of astroglial Ca^2+^-signaling on firing rate or plasticity of neural networks cannot be *a priori* considered as solely positive (or negative). This might explain why some attempts to genetically modify Ca^2+^ signaling in astrocytes did not yield the expected results (Bazargani and Attwell, 2016; Fiacco and McCarthy, 2018; Savtchouk and Volterra, 2018). Our data also suggest that release of gliotransmitters, in particular ATP and D-Serine, can be affected by environment, physical exercise and diet, in addition to the recently reported effects of sleep cycle (Papouin et al., 2017a). A high degree of experience- and environment-related plasticity of astroglial function may further add to the variability of the results obtained in different laboratory settings.

Our results, in particular shown in Figure 2, can also be important for the current debate on mechanisms D-Serine release in the brain. Although a large body of evidence, obtained in the last decade using different experimental approaches supports an importance of astroglia in the release of D-Serine and in the D-Serine-mediated modulation of NMDA receptors, an alternative (and rather extreme) view of predominantly neuronal release of D-Serine at physiological conditions has been recently suggested (Wolosker et al., 2016). One should note that notion of predomimant role of neurons in release of D-Serine originates mainly from the data suggesting a high level of expression of SRR in neurons rather than astroglia. However, this contradicts to recent transcriptomic data showing relatively high level of *Srr* gene expression in cortical and hippocampal astrocytes (Chai et al., 2017). There are several other flaws in the “neuronal D-Serine” theory which have been thoroughly addressed by Papouin et al. (2017b).

One should emphasize that even the studies, questioning astroglia-specific expression of serine racemase, do not deny an ability of astrocytes to accumulate D-serine (Wolosker et al., 2016) and a reconciliating theory of cooperation between neuronal and glial release has been recently suggested by Ivanov and Mothet (2018). Whatever is concentration of D-Serine in neurons and astrocytes, their contribution into extracellular D-serine level will depend mainly on efficiency of neuronal vs. glial mechanisms of release. So far, there is a lack direct evidence that neurons possess an efficient mechanism of release of D-serine (Papouin et al., 2017b), with Asc-1 being suggest to be a main pathway (Papouin et al., 2017b). Release of D-serine from strocytes can occur via Ca^2+^-dependent exocytosis, as an alternative pathway one might also suggest Ca^2+^-dependent large conductance chloride channels (Woo et al., 2012). These channels have been shown to be permeable to glutamate (Woo et al., 2012) which has even larger molecular size than D-Serine.

Compared to vesicular or channel-mediated release allowing the movement of multiple molecules, transporter, that releases a single molecule per single act of conformational change, is intrinsically slow. So, one could hardly expect the putative Asc-1 transporter-mediated release of D-Serine from neurons to be more than Ca^2+^-dependent release of D-Serine from astrocytes.

Indeed, our experiments in isolated neurons and neuron-astrocyte “bundles,” which allowed to dis-entangle glial and neuronal sources of D-Serine, showed that neuronal release on its own could not provide enough D-Serine to maintain the activity of synaptic NMDAR (Figure 2). We would like to stress that our preparation of isolated neurons provide a very good accessibility of synapses to D-amino acid oxidase, however we did not observe any marked effect of DAAO on synaptic NMDAR-mediated currents in conditions when neurons were perfused with 1 mM D-Serine via whole-cell recording pipette. This observation on its own argues against efficient release of D-Serine from neurons. Combined with results of biosensor measurements of D-serine release in brain slices of WT and dnSNARE mice (Figure 1D, see also Rasooli-Nejad et al., 2014), our data strongly support an importance of astrocytes as a source of D-serine at physiological conditions.

Our data on age-related changes in astroglial Ca^2+^-signaling and release of ATP and D-Serine (Figure 1) go in line with accumulating evidence that alterations in extracellular levels of ATP, adenosine and D-Serine can play an important role in physiological and pathological brain aging (Mothet et al., 2006; Orellana et al., 2012; Gundersen et al., 2015; Rodrigues et al., 2015; Piacentini et al., 2017). Our present (Figures 2, 3) and previous data (Lalo et al., 2014b, 2016; Pankratov and Lalo, 2015) on astroglial-driven modulation of synaptic transmission and plasticity supports the recently emerged view that molecular and functional alteration in astrocytes, such as release of gliotransmitters, can precede or even cause, changes in synaptic dynamics and homeostasis during aging and progression of neurodegenerative diseases (De Strooper and Karran, 2016; Soreq et al., 2017). Our results also suggest that widely reported beneficial effects (Hillman et al., 2008; Nithianantharajah and Hannan, 2009; van Praag, 2009; Mercken et al., 2012; Merzenich et al., 2014) of enriched environment on synaptic plasticity and memory in mice and, possibly, effects of active life style in humans, can be mediated by enhancement of release of gliotransmitters, very likely due to increased endocannabinoid and adrenergic Ca^2+^-signaling in astrocytes. Since both EE and CR upregulated Ca^2+^signaling in astrocytes and affected synaptic plasticity in the dnSNARE-dependent manner, one can hypothesize that beneficial effects of CR on synaptic plasticity also originate from enhanced gliotransmission and glia-neuron communications. Still, alternative mechanisms of action of CR might be suggested, which can be underlined by various metabolic effects of CR (Mercken et al., 2012; Park et al., 2012; Madeo et al., 2014; López-Otín et al., 2016), for example enhancement mitochondrial function in astrocytes. Specific roles for gliotransmission- and metabolism-related mechanisms in beneficial effects of CR on aging brain are yet to be studied.

To conclude, our results strongly support physiological importance of astroglial cannabinoid and adrenergic signaling and glia-derived ATP for communication between astrocytes and neurons and experience-related modulation of synaptic plasticity across a lifetime.

## Author Contributions

UL, AB and YP contributed to the design and interpretation of experiments and commented on the manuscript. UL and AB performed experiments and data analysis. YP and UL conceived the study and wrote the manuscript.

## Conflict of Interest Statement

The authors declare that the research was conducted in the absence of any commercial or financial relationships that could be construed as a potential conflict of interest.
